# Metabolomics in juvenile idiopathic arthritis: A distinct profile in patients under methotrexate

**DOI:** 10.1016/j.clinsp.2024.100522

**Published:** 2025-01-28

**Authors:** Renato B. Tomioka, Gabriela R.V. Ferreira, Nadia E. Aikawa, Gustavo A.R. Maciel, José M. Soares Junior, Edmund C. Baracat, Eloisa Bonfá, Ismael Dale Cotrim Guerreiro da Silva, Clovis Almeida da Silva

**Affiliations:** a Division of Rheumatology, Hospital das Clínicas, Faculdade de Medicina da Universidade de São Paulo (HCFMUSP), São Paulo, SP, Brazil; b Discipline of Gynecology, Hospital das Clínicas, Faculdade de Medicina da Universidade de São Paulo (HCFMUSP), São Paulo, SP, Brazil; c Pediatric Rheumatology Unit, Hospital das Clínicas, Faculdade de Medicina da Universidade de São Paulo (HCFMUSP), São Paulo, SP, Brazil; d Departament of Gynecology, Escola Paulista de Medicina, Federal University of São Paulo, São Paulo, SP, Brazil

**Keywords:** Juvenile idiopathic arthritis, Metabolomics, Biomarkers, methotrexate (Methotrexate), Juvenile idiopathic arthritis subtypes

## Abstract

The objective of the present study was to evaluate biochemical quantitative metabolites in peripheral blood serum samples of Juvenile Idiopathic Arthritis (JIA) patients and healthy controls. A cross-sectional study included 33 post-pubertal JIA (21 without and 12 with Methotrexate (MTX) women and 28 age-matched healthy controls. Metabolomic analyses based on targeted electrospray ionization tandem mass spectrometry were used to identify possible biochemical pathway modifications in serum from JIA patients. The mean current age (*p* = 0.065) was similar in JIA patients and healthy controls. Current MTX use in all subtypes of JIA patients was associated with an increase in concentrations of free carnitine [21.74 (12.7‒35.2) vs. 27.49 (14.5‒41.3) µM/L, *p* = 0.02], suggesting an enhanced mitochondrial metabolism and intestinal absorptive function. In contrast, a decreased mitochondrial metabolism was observed in polyarticular and systemic JIA subtypes, with a decrease of several acylcarnitines’ concentrations (*p* < 0.05). In conclusion, the present study identified a distinctive pattern of serum metabolic signatures in JIA patients under MTX therapy. Our findings indicate that MTX use is associated with a more efficient mitochondrial function.

## Highlights

• We identified a distinctive pattern of serum metabolic signatures in juvenile idiopathic arthritis patients under methotrexate therapy.
• Methotrexate use is associated with a more efficient mitochondrial function.
• A decreased mitochondrial metabolism was observed in polyarticular and systemic JIA subtypes, with a decrease of several acylcarnitines’ concentrations.

## Introduction

Juvenile Idiopathic Arthritis (JIA) includes a group of chronic arthritis that affects individuals younger than 16 years of age.^1^^,^^2^ JIA is an umbrella term for seven different subtypes of chronic arthritis and is classified by the International League of Associations for Rheumatology (ILAR) criteria in systemic arthritis; polyarthritis Rheumatoid Factor (RF)-positive, polyarthritis RF-negative, oligoarthritis (persistent or extended), enthesitis-related arthritis, psoriatic arthritis, and undifferentiated arthritis.^3^ This rare condition presents various clinical and laboratory phenotypes, including potential biomarkers.^1^^,^^4^^,^^5^ Biomarkers are an important component of personalized medicine and have diagnostic, prognostic, or therapeutic utilities.^6^ Serum biomarkers have shown a potential to identify different clinical phenotypes, disease activity and severity, and to predict treatment response in JIA patients.^7^^,^^8^

In this regard, proteomics has identified that serum levels of matrix metalloproteinase-3 are high in JIA patients, and seem to be a biomarker of disease activity.^9^, ^10^, ^11^ Myeloid-related protein 8/14 and the neutrophil-derived S100A12 were also reported to be biomarkers of JIA disease activity and to predict a subgroup of patients who will improve after Methotrexate (MTX) use.^12^^,^^13^

Metabolomics analyses are scarce and seem to provide valuable information on the metabolite's dysfunction in adults with ARDs.^14^ In fact, this technique detected changes in the concentration of small endogenous metabolites in biological samples of adult patients with Rheumatoid Arthritis (RA).^15^ and of potential importance were metabolic events of glycolysis and amino acid metabolism that seemed to be related to MTX response.^16^

Analysis of metabolomics in JIA patients is restricted to a few case reports with one patient^17^ and another focusing on a restricted cluster of lipoproteins measurements.^18^ The use of untargeted mass spectrometry^19^ in one study, hampers the comparison of various biochemical quantitative metabolomics simultaneously in patients with and without MTX use.

Therefore, the objectives of the present study were to evaluate biochemical quantitative metabolites in peripheral blood serum samples of JIA female patients compared to healthy controls. In addition, the metabolomic profile under MTX treatment was evaluated in these patients.

## Material and methods

### Ethical aspects

The study was approved by the Local Ethics Committee of the Hospital das Clinicas HCFMUSP, Faculdade de Medicina, Universidade de São Paulo, São Paulo, SP, BR, and all authors, or their legal guardians, provided written informed consent.

### Case-control setting

Patient selection included 34 consecutive post-pubertal female JIA patients, with ages ranging from 15 to 38 years. All JIA patients met the disease classification criteria set by the ILAR[3] and were monitored at the Rheumatology Division or Pediatric Rheumatology Unit of our tertiary center. One JIA patient was excluded for technical issues, due to the degradation of the collected sample. Thus, 33 JIA patients comprised the study group. A healthy control group balanced by age included 28 post-pubertal females, between 15 and 35 years of age.

All blood samples of JIA patients and controls were collected at the beginning of the menstrual cycle (at the follicular phase) in Ethylenediamine Tetraacetic Acid (EDTA) containing tubes. After sample centrifugation at 1000 × *g* for 10 min and transferred to a new tube, serum samples were stored immediately at −80 °C until analysis.

### Demographic, clinical, laboratorial and treatment assessments

Age, Body Mass Index (BMI), and smoking status were systematically evaluated. BMI was calculated as weight in kilograms divided by the square of the body height (kg/m²). In order to determine the JIA disease activity, the following parameters were evaluated: the number of active and limited joints (determined by the presence of joint edema or the presence of at least two of the following: joint pain on mobilization, joint tenderness, or joint limitation), global assessment of arthritis activity by both the patient and physician using a 10 cm horizontal Visual Analogue Scale (VAS), where 0 indicated no activity and 10 indicated maximum activity. C-Reactive Protein (CRP) levels were measured by nephelometry, and Erythrocyte Sedimentation Rate (ESR) was measured using the Westergren method.

To determine the disease activity, the Juvenile Arthritis Disease Activity Score (JADAS-71) (range: 0‒101 points) was used for patients up to 18 years,^20^ and the Disease Activity Score 28-Joint Counts (DAS-28) was used for patients with >18 years.^21^

Use of MTX and other disease-modifying anti-rheumatic drugs, such as DMARDs (leflunomide, sulfasalazine, and hydroxychloroquine), glucocorticoid, and biological agents (etanercept, adalimumab, golimumab, certolizumab pegol, abatacept and tocilizumab) was also recorded.

### Metabolomic profiling

Serum samples preserved at a temperature of −80 °C were maintained in dry ice during transportation. The absolute quantification (µM/L) of metabolites in peripheral blood was accomplished through targeted quantitative analysis of 186 metabolites using Electrospray Ionization (ESI) tandem mass spectrometry (MS/MS) in the serum samples. This analysis was conducted on the SCIEX 5500 QTRAP instrument (SCIEX, Darmstadt, Germany), blinded to any phenotypic information. For that, the quantitative metabolomics platform provided by BIOCRATES Life Sciences AG, Innsbruck, Austria (https://biocrates.com/), was used on a centralized, independent, fee-for-service basis.

The patent US 2007/0,004,044 (available online at www.freepatentsonline.com/20,070,004,044.html) provides a comprehensive description of the experimental approach used for metabolomics measurements. In summary, a targeted profiling scheme was employed to quantitatively examine fully annotated metabolites using various techniques such as multiple reaction monitoring, neutral loss, and precursor ion scans. The MetIQ software package (BIOCRATES Life Sciences AG, Innsbruck, Austria) was employed for both metabolite concentration quantification and quality control assessment, following the 21CFR (Code of Federal Regulations) Part 11 guidelines, which ensures reproducibility within an acceptable error range. Subsequently, an Excel (xls) file was generated, encompassing sample identification along with the names and concentrations of 186 metabolites presented in μM/L of serum (https://biocrates.com/).

The present study used a targeted approach that included four main metabolic areas: amino acids (and biogenic amines), glucose, fatty acids (acylcarnitines), and lipid metabolism (glycerophospholipids and sphingolipids). A total of 186 annotated metabolites were quantified using the AbsoluteIDQ® p180 kit (BIOCRATES Life Sciences AG, Innsbruck, Austria): 21 Amino Acids (AAs); 19 Biogenic Amines (BA); 40 Acylcanitines (ACs); 1 sum of Hexoses (Hex), which is vastly represented by glucose (up to 95 %); 76 Phosphatidylcholines (PCs); 14 Lyso-Phosphatidylcholines (LPCs) and 15 Sphingomyelins (SMs). Glycerophospholipids were distinguished based on the type of bond present in the glycerol moiety, specifically ester (a) and ether (e). For the notation, two letters were used to indicate that two glycerol positions were linked to a fatty acid residue (aa = diacyl, ae = acyl-alkyl), while a single letter indicated the presence of a single fatty acid residue (*a* = acyl or *e* = alkyl) (https://biocrates.com/). Thus, the biochemical analyses provided results referring to at least 5 classes of metabolites with structures, function, and blood concentrations, previously established in healthy subjects, which are widely and routinely used in clinical laboratories around the world, as an established tool for the diagnosis of the inborn errors of metabolism.^22^

### Metabolite set enrichment analysis (MSEA)

Data obtained from targeted quantitative Electrospray Ionization Tandem Mass Spectrometry (ESI-MS/MS) were uploaded onto MSEA, an unsupervised tool available at www.metaboanalyst.ca, that assists in the identification of biochemical disturbances found in patients with JIA.

### Statistical analyses

Statistical analyses were conducted using the Statistical Package for the Social Sciences, version 24.0 for Windows. Results for the continuous variables were reported as either median (with minimum and maximum values) or mean ± Standard Deviation (SD), while categorical variables were presented as frequency (percentage). To assess the homogeneity of variances, Levene's test was performed. Scores with a normal distribution were compared using Student's *t*-test, whereas those with abnormal distributions were assessed using the Mann-Whitney test. Differences in categorical variables were calculated using either Fisher's exact test or Pearson's Chi-Square test, as appropriate.

To analyze the metabolomic data, a log transformation was used to normalize the concentration distributions of the quantified metabolites. These transformed data were then uploaded to the web-based analytical pipelines of MetaboAnalyst 5.0 (www.metaboanalyst.ca).^23^ The analysis of variance (ANOVA) was employed to compare the variables among the three groups, followed by a post hoc analysis using Dunn's multiple comparison test to identify specific differences between the groups. Pearson's “*r*” analysis was employed to assess the extent of linear correlation between two quantitative variables. The significance level for all analyses was set at 5 %.

## Results

### Demographic, clinical, laboratorial and treatment assessments

The mean current age (23.15 ± 6.34 vs. 26.14 ± 6.03 years, *p* = 0.065) was similar in JIA patients and healthy controls. No differences were observed regarding the mean BMI (23.04 ± 3.18 vs. 23.26 ± 3.27 kg m^-12^, *p* = 0.797) and the frequency of smoking (12 % vs. 3 %, *p* = 0.363) in both groups (Table 1).

**Table 1:** Clinical and laboratorial parameters, and concomitant treatments in Juvenile Idiopathic Arthritis (JIA) patients with and without current use of Methotrexate (MTX).

**Variables**	**With MTX** **(*n* = 12)**	**Without MTX** **(*n* = 21)**	***p***
**Clinical and laboratorial parameters**			
Number of active joints	0.5 (0–10)	0.5 (0–10)	0.868
Number of limited joints	4 (0–32)	6 (0–43)	0.427
Patient global VAS (0–10)	3 (1–9)	1 (0–6)	**0.027**
Physician global VAS (0–10)	1 (0–2)	1 (0–5)	0.449
ESR, mm/1st hour	11.25 ± 7.67	11.67 ± 9.42	0.897
CRP, mg/L	4.17 ± 4.98	6.76 ± 16.51	0.602
JADAS-71	5.00 ± 0.00	1.57 ± 2.63	0.123
DAS-28	2.40 ± 1.17	2.36 ± 1.00	0.924
**Current treatment**			
Prednisone	2 (16.7)	4 (19)	1.000
Leflunomide	2 (16.7)	7 (33.3)	0.429
Hydroxychloroquine	1 (8.3)	0 (0)	0.364
Biologic agent use	7 (58.3)	12 (57.1)	1.000
**Biological agent's types**			
Etanercept	4 (33.3)	4 (19 %)	0.420
Adalimumab	1 (8.3)	1 (4.8)	1.000
Golimumab	1 (8.3)	0 (0)	0.364
Certolizumab pegol	0 (0)	1 (4.8)	1.000
Abatacept	0 (0)	2 (9.5)	0.523
Tocilizumab	1 (8.3)	4 (19)	0.630

Values expressed as n (%), median (minimum and maximum values) or mean ± standard deviation; VAS, Visual Analog Scale; ESR, Erythrocyte Sedimentation Rate; CRP, C-reactive Protein; JADAS-71, Juvenile Arthritis Disease Activity Score; DAS-28, Disease Activity Score 28-Joint Counts.

According to disease subtypes, polyarticular JIA occurred in 21/33 (63.7 %) patients, systemic JIA in 7/33 (21.2 %), oligoarticular JIA in 4/33 (12.1 %) and enthesitis-related arthritis JIA in 1/33 (3 %).

Table 1 illustrates clinical and laboratorial parameters, and concomitant treatments in JIA patients with and without the current use of MTX. Patient global VAS was significantly higher in JIA patients with MTX in comparison to those that did not receive the treatment [3 (1‒9) vs. 1 (0‒6), *p* = 0.027]. Clinical and laboratorial disease parameters, such as a number of active and limited joints, physician global VAS, ESR, CRP, JADAS-71 and DAS-28 were similar in both groups (*p* > 0.05). No differences were observed regarding the use of prednisone, leflunomide, hydroxychloroquine and biological agents between both JIA groups (*p* > 0.05) (Table 1).

### Conditions that presented similar biochemical phenotypes with JIA

**Fig. 1:**
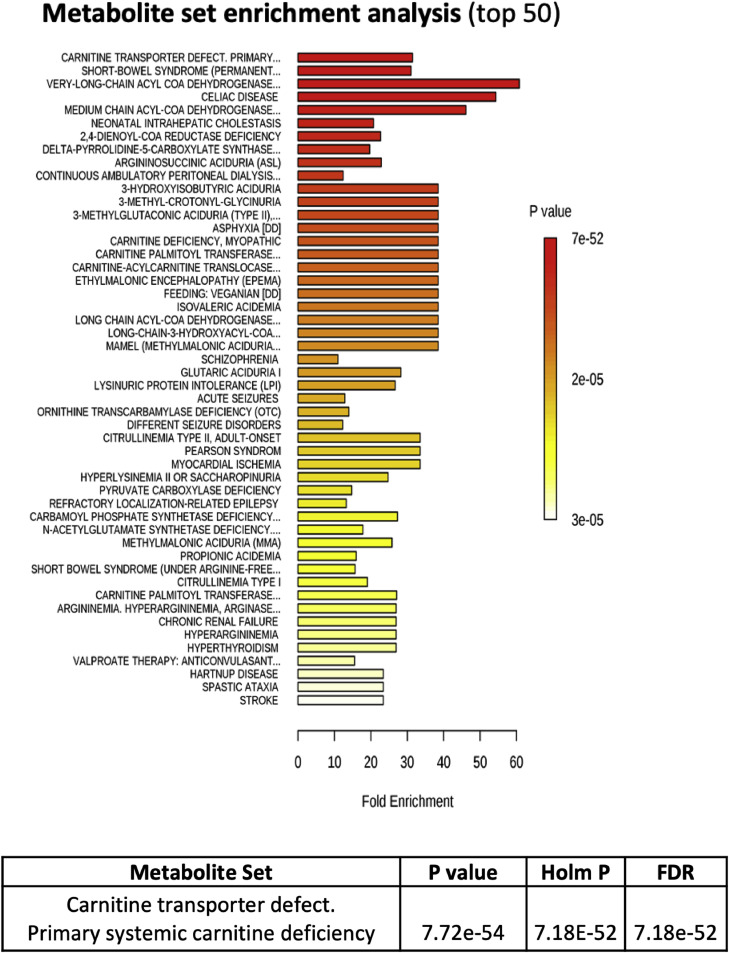
Quantitative functional enrichment analysis after uploading the quantitative metabolites of juvenile idiopathic arthritis (JIA) patients to the metabolic set enrichment analysis (MSEA) with top 50 conditions with similar biochemical phenotypes than JIA. Color intensity (white to red) indicates increasing statistical significance.

The quantitative results of metabolites were entered into specific bioinformatics software for metabolomic analysis available by the University of Alberta (Canada) at www.metaboanalyst.ca. From this perspective, the study was initiated using the quantitative functional enrichment tool (MSEA) where, in an unsupervised way, the biochemical results obtained are compared with a metabolomic database containing biochemical information on hundreds of human metabolic conditions. Finding similarities with some dysfunctions, the results were arranged in decreasing order of statistical significance as shown in Fig. 1. Thus, functional enrichment in red demonstrated the disturbances that were most associated with the values generated by quantitative mass spectrometry performed on samples of JIA patients and controls. The most significant results considering a highly demanding cutoff of 10e-52 revealed 5 conditions with high chances of belonging to involved metabolic pathways, which in the present study seem to overlap in their origin. The five conditions that presented similar biochemical phenotypes with JIA were: carnitine transport defects, short bowel syndrome, deficiencies in very-long and medium chain acyl-CoA dehydrogenases, and celiac disease.

Current MTX use in JIA patients is associated with an increase in free carnitine's concentrations, suggesting an improved mitochondrial metabolism and intestinal absorptive function.

**Fig. 2:**
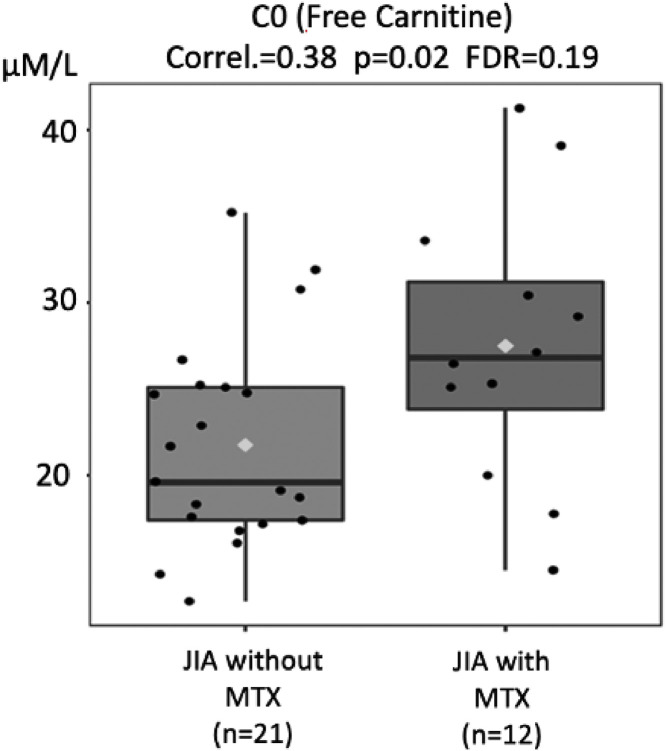
Comparison of free carnitine molar levels (Y-axis) in juvenile idiopathic arthritis (JIA) patients without and with current use of methotrexate (MTX) (X-axis). FDR - false discovery ratio (Pearson's correlation analysis).

Fig. 2 shows the values of free Carnitine (C0) detected in the circulation of JIA patients with and without MTX. JIA patients without current MTX use had significantly lower levels of C0 compared to the JIA with MTX [21.74 µM/L (12.7‒35.2) vs. 27.49 µM/L (14.5‒41.3), *p* = 0.02]. It is important to note that C0 levels in the healthy population in the literature range from 25 to 50 µM/L.^24^ Defects in carnitine transport are biochemical deviations with primary causes (inherited mutations in intestinal transporters, such as inborn errors of metabolism) or secondary causes, particularly related to intestinal absorption deficiencies of other origins, such as short bowel syndrome and celiac disease.^25^, ^26^, ^27^ These dysfunctions suggested in mitochondrial dehydrogenases are part of a disorder secondary to low concentrations of C0, leading to low formation of acylcarnitines with a consequent decrease in mitochondrial function in JIA patients without MTX.

### Current MTX use in JIA patients is associated with increased acylcarnitines’ concentrations

**Fig. 3:**
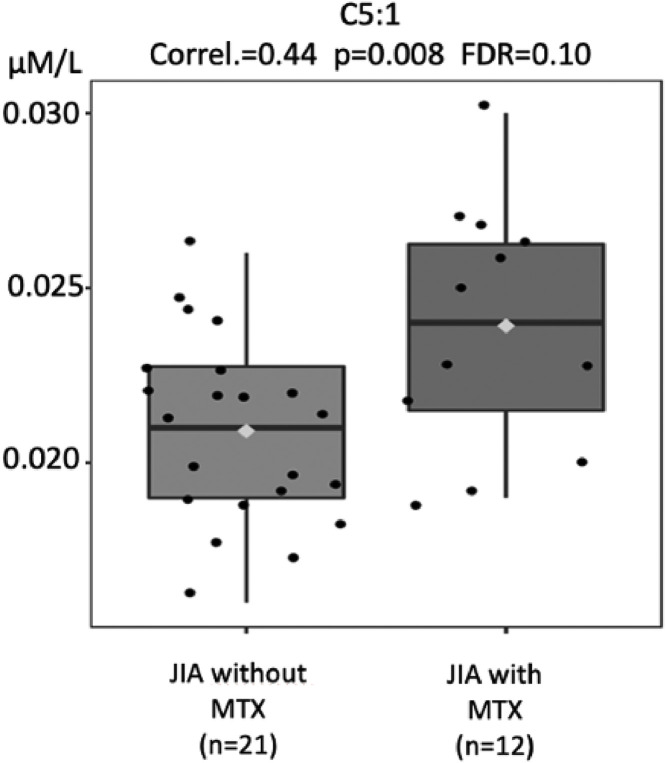
Comparison of tiglylcarnitine (C5:1) molar levels (Y-axis) in JIA patients with and without current use of methotrexate (MTX) (X-axis). FDR - false discovery ratio (Pearson's correlation analysis).

**Fig. 4:**
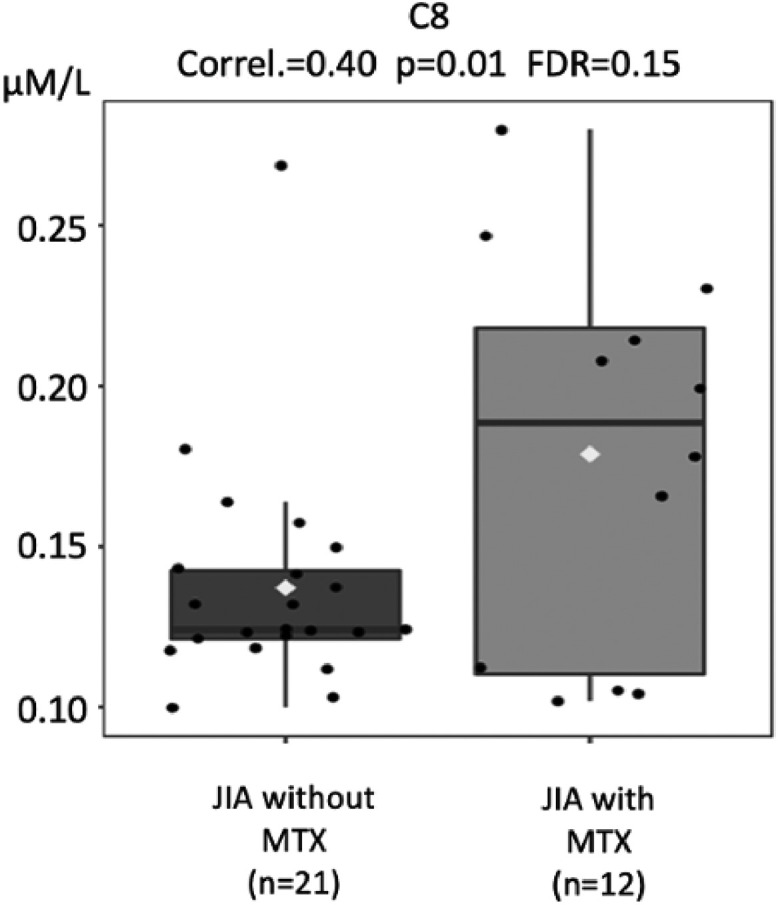
Comparison of octanoylcarnitine (C8) molar levels (Y-axis) in JIA patients with and without current use of methotrexate (MTX) (X-axis). FDR - false discovery ratio (Pearson's correlation analysis).

**Fig. 5:**
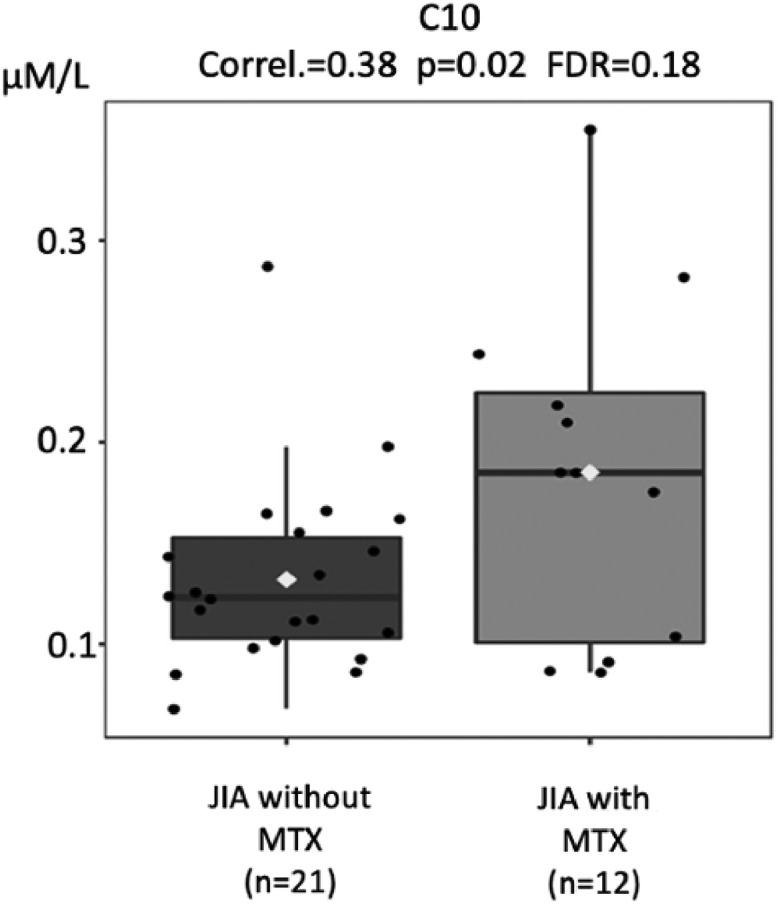
Comparison of decanoylcarnitine (C10) molar levels (Y-axis) in JIA patients with and without current use of methotrexate (MTX) (X-axis). FDR - false discovery ratio. FDR: false discovery ratio (Pearson's correlation analysis).

**Fig. 6:**
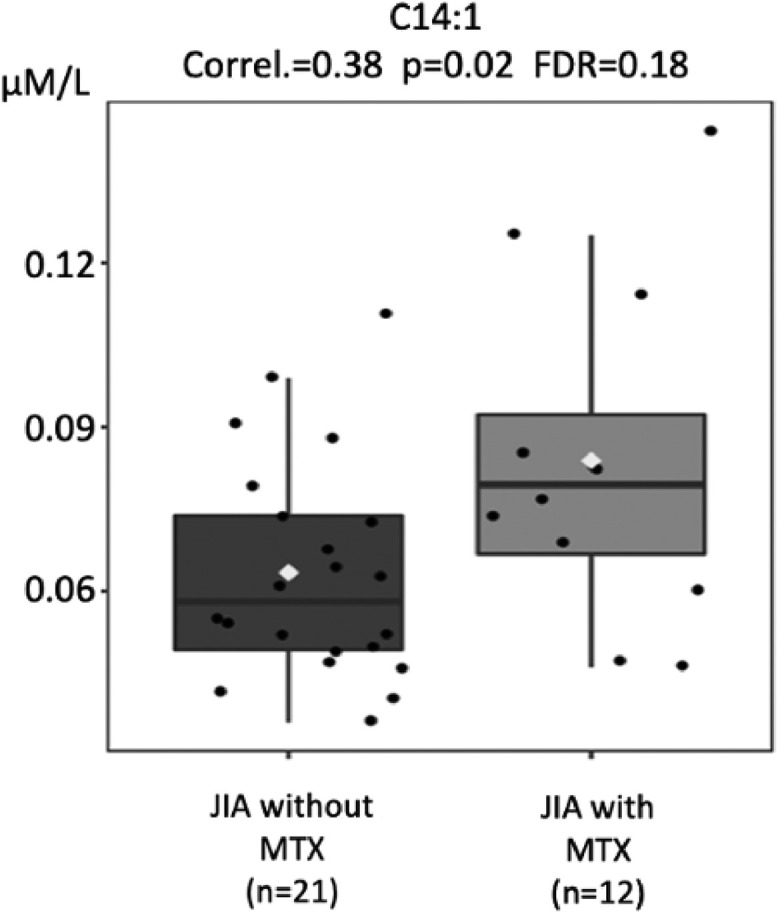
Comparison of tetradecenoylcarnitine (C14:1) molar levels (Y-axis) in JIA patients with and without current use of methotrexate (MTX) (X-axis). FDR - false discovery ratio (Pearson's correlation analysis).

Acylcarnitine levels are known to be low in patients with impaired absorption of free carnitine.^25^^,^^28^ Based on the biochemical alterations found in carnitine deficiencies, it is classically known that these metabolic alterations lead to a decrease in the blood profile of all acylcarnitines, whether they are carriers of small, medium, or large chain fatty acids. Thus, analyses of acylcarnitines were performed in the three groups, shown in Fig. 3, Fig. 4, Fig. 5, Fig. 6.

The levels of different acylcarnitines were also significantly lower in JIA patients without current use of MTX compared to those with MTX, such as tiglylcarnitine (C5:1; *p* = 0.008), octanoylcarnitine (C8; *p* = 0.01), decanoylcarnitine (C10; *p* = 0.02), tetradecenoylcarnitine (C14:1, *p* = 0.02), that is from short, medium to long acylcarnitines (Fig. 3, Fig. 4, Fig. 5, Fig. 6).

### JIA patients showed biochemical dysfunctions, indicating disorder in the metabolism of structural lipids, asymmetric dimethylarginine and demethylated arginine

**Fig. 7:**
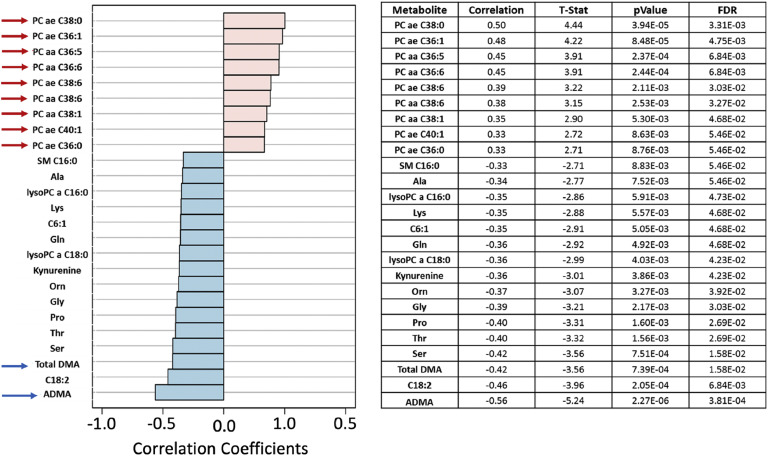
Pearson “r” type correlation analysis illustrates metabolites positively (pink) and negatively (blue) correlated with the phenotypes involving juvenile idiopathic (JIA) without methotrexate (MTX), JIA with MTX and healthy controls. Positive correlations with elevated concentrations of structural lipids, such as phosphatidylcholines diacyl (PC aa) and acyl-alkyl (PC ae) (red arrows), were observed in controls compared to JIA with and without MTX current use.

**Fig. 8:**
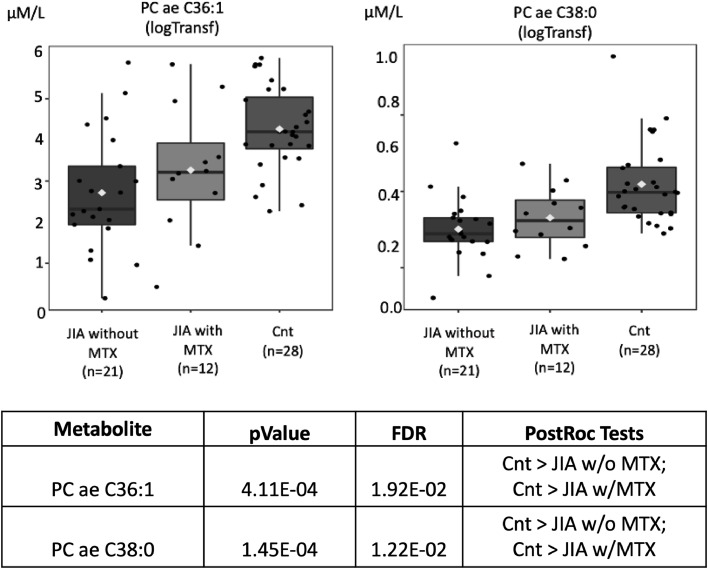
Phosphatidylcholine with acyl-alkyl residue sum C36:1 (PC ae C36:1) and Phosphatidylcholine with acyl-alkyl residue sum C38:0 (PC ae C38:0) molar levels (Y-axis) in juvenile idiopathic arthritis (JIA) patients with and without current use of methotrexate (MTX) and healthy controls (Cnt) (X-axis). FDR - false discovery ratio; JIA w/o MTX - JIA without current MTX use; JIA w MTX - JIA with current MTX use (analysis of variance - ANOVA).

The possibility of absorptive dysfunction being associated with low lipid levels was explored. In Fig. 7, the Pearson “*r*” type correlation analysis illustrated positive (pink) and negative (blue) metabolites correlated with the phenotypes involving three groups: JIA without MTX, JIA with MTX and healthy controls. Positive correlations were evidenced with structural lipids such as Phosphatidylcholines diacyl (PC aa) and acyl-alkyl (PC ae) (red arrows) which have their concentrations elevated in JIA with MTX compared to controls. The levels of Phosphatidylcholine with acyl-alkyl residue sum C36:1 (PC ae C36:1) and Phosphatidylcholine with acyl-alkyl residue sum C38:0 (PC ae C38:0) were significantly higher in healthy controls compared to JIA with or without MTX (*p* = 4.11E-04 and *p* = 1.45E-04, respectively) (Fig. 8).

**Fig. 9:**
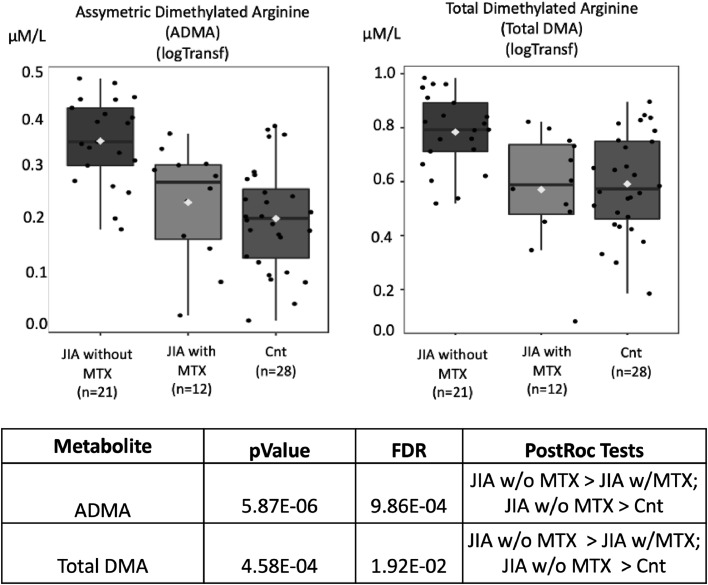
Asymmetric dimethylarginine (ADMA) and total demethylated arginine (DMA) molar levels (Y-axis) in juvenile idiopathic arthritis (JIA) patients with and without current methotrexate (MTX) use and healthy controls (Cnt) (X-axis). FDR - false discovery ratio; JIA w/o MTX - JIA without current MTX use; JIA w MTX - JIA with current MTX use (analysis of variance - ANOVA).

On the other hand, decreases in the levels of systemic dimethylation of arginine residues (Asymmetric Dimethylarginine ‒ ADMA and total Dimethylamine ‒ DMA) (blue arrows) were shown in healthy controls than JIA patients with and without MTX. Asymmetric Dimethylarginine (ADMA) serum levels were higher in JIA without MTX versus JIA with MTX versus healthy controls (*p* = 5.87E-06; FDR = 9.86E-04). Total Demethylated Arginine (DMA) levels were also higher in JIA without MTX versus JIA with MTX versus controls (*p* = 4.58E-04; FDR = 1.92E-02) (Fig. 9).

## Discussion

We demonstrated a distinct serum metabolic profile using a targeted quantitative metabolomic approach in JIA patients. This metabolomic pattern was characterized by lower molar levels of free carnitine, acylcarnitines and lipids, with higher concentrations of asymmetric dimethylarginine and demethylated arginine in patients without MTX therapy.

The main strength of the present study was the use of a completely validated metabolomic tool in post-pubertal JIA women and healthy female controls, with the standardization of blood samples after 8 to 12 h of the fasting period. The samples were also collected at the follicular phase of the menstrual cycle since metabolic changes can be evidenced among different cycle phases.^29^ Another advantage of this study, was the use of a metabolomic targeted approach that enables a correct identification of metabolites and the precise calculation of their concentrations, contrasting with a previous report that used an untargeted method in JIA patients.^19^ Female JIA patients and controls lower than 40 years and with similar BMI and smoking frequency were also relevant, since sex, age, smoking, overweight and obesity may influence metabolic biomarkers.^30^^,^^31^ We also excluded patients who had a difficult venous puncture, leading to material degradation, that could induce errors in the metabolite's quantification.^32^ A possible limitation of our study is the small sample size due to disease rarity and the cross-sectional design, precluding the generalization of the results.

We extended previous studies identifying novel serum biomarkers and metabolic signatures with the expression of free carnitine and acylcarnitines in JIA patients under MTX therapy.^17^, ^18^, ^19^ Our findings showed metabolic disorders resembling mitochondrial disturbances as underlying conditions. Indeed, carnitine has an essential role in the transfer of long-chain fatty acids across the inner mitochondrial membrane, where they are metabolized via β-oxidation.^33^ Despite serving as a critical screening test for inborn errors of metabolism in newborns, the measurement of free carnitine is not usual in the adult population.^34^ Nowadays, free carnitine and acylcarnitines serve as potential biomarkers for illness and drug response and are also known for participating in Fatty Acid β-Oxidation (FAO) in mitochondria and peroxisomes.^28^ Disruption in mitochondrial function is related to a myriad of acute and chronic diseases, such as cancer, sepsis, cardiovascular and immunological diseases.^35^

MTX remains the main widely used conventional DMARD and is the most effective non-biologic agent for JIA patients.^36^ This medication is primarily recognized for its potent inhibition of folate metabolism, affecting various Targets involved in one-carbon metabolism, nucleotide synthesis, and amino acid biosynthesis.^37^ MTX acts by inhibiting Aminoimidazole-4-Carboxamide Ribonucleotide (AICAR) transformylase, leading to the accumulation of adenosine within the cells, which then binds to cell surface receptors and suppresses numerous inflammatory and immune responses.^38^ Importantly, concentrations of specific metabolites differed between RA patients receiving MTX treatment and those without, consistent with previous findings suggesting a potential correlation between underlying metabolic events and the response to MTX in early adult RA.^16^

The lower levels of free carnitine and acylcarnitines in JIA patients without MTX may reflect a dysfunction in the intestinal absorption of carnitine, with consequent worsening of mitochondrial function and FAO. Therefore, we hypothesize that free carnitine values approaching normal levels[24] in JIA patients using MTX observed herein, is probably a consequence of an improvement in the biochemical picture through the anti-inflammatory and immunomodulatory effects of this drug.^38^ In fact, higher levels of acylcarnitines in patients under MTX corroborate with the concept of a better mitochondrial function in JIA patients using this drug. Indeed, JIA patients may present energy metabolism abnormalities, probably due to mitochondrial dysfunction.^39^, ^40^, ^41^

In the present study, we demonstrated that JIA patients showed biochemical dysfunctions indicating disorder in the metabolism of structural lipids in accordance with lipid changes described previously in JIA patients,^42^, ^43^, ^44^ which are associated with higher cardiovascular risk in these patients.^45^

Sphingomyelins (SM) consist of a ceramide core connected to a single fatty acid and to a phosphocholine or phosphoethanolamine. Alongside Phosphatidylcholines (PC), these phospholipids are the main components of cellular membranes and can participate in cellular signal transduction.^46^ They constitute a significant proportion of the lipidome found in human plasma since they are highly abundant in all lipoproteins.^47^ The metabolism of phospholipids plays a crucial role in regulating lipid, lipoprotein, and mitochondrial energy production, which is associated with disease progression.^48^ In fact, major metabolic pathways that are inhibited by MTX therapy in JIA patients include lipid metabolism, with the inhibition of arachidonic acid metabolism and changes in the cellular lipid composition noted in vitro. These findings suggest that MTX influences cellular lipid homeostasis, resulting in a decrease in saturated Fatty Acids (FA)-containing lipids, reduced cholesterol esters, and an increase in unsaturated FA-containing lipids.^49^ This mechanism leads to beneficial cardiovascular effects and, indeed, MTX therapy in the management of autoimmune arthritis is associated with a reduction in cardiovascular risk.^50^

Moreover, circulating pro-/anti-inflammatory metabolic signatures indicating disease activity and inflammatory status were reported in RA patients.^15^ These findings were not observed in our JIA patients, possibly because most patients were under DMARDs and biologic agents and had controlled disease.

Increased serum concentrations of ADMA, an endogenous inhibitor of Nitric Oxide Synthase (NOS), have been reported in several rheumatic diseases, such as systemic lupus erythematosus, psoriatic arthritis, Sjogren's syndrome, Behçet's disease, ankylosing spondylitis, and systemic sclerosis, supporting a potential pathogenetic role for the accumulation of ADMA in the occurrence and progression of endothelial dysfunction, arterial hardening, and atherosclerosis.^51^ We have shown higher levels of ADMA in the JIA without MTX group, which may be related to the anti-inflammatory and cardioprotective effect of MTX.^38^^,^^52^

Further longitudinal studies are necessary to define if the metabolomic profile of JIA under MTX identified herein will be a valuable biomarker for treatment response in patients with this condition.

## Conclusion

The present study identified novel serum metabolic signatures in JIA patients under MTX therapy. Our findings strongly suggest that mitochondrial function disturbances improved with MTX use.

## Authorship criteria

All authors approved the final version of the manuscript, agreed with its submission, and took full responsibility for all information provided.

## Ethics committee name and study protocol number

CAAE number 33,275,214.8.0000.0068

## Authors’ contributions

All authors made significant contributions to the conception and design of the study, as well as the analysis and interpretation of the data. Additionally, all authors thoroughly reviewed the work, provided critical feedback, and gave their approval for the final version.

## Funding

This study was supported by grants from Fundação de Amparo à Pesquisa do Estado de São Paulo (FAPESP 2014/14806–0 to CAS; #2015/03756–4 to CAS and EB), Conselho Nacional de Desenvolvimento Científico e Tecnológico (CNPq 306864/2021–5 to RMRP, 305068/2014–8 to EB and **303,422/2015–7 to CAS**)), Federico Foundation (to RMRP, EB and CAS) and by Núcleo de Apoio à Pesquisa “Saúde da Criança e do Adolescente” da USP (NAP-CriAd) to CAS.

## Financial disclosure

The authors do not have any financial agreement with a company whose product is prominently featured in the present manuscript or with a company that produces a competing product.

## Conflicts of interest

The authors declare no conflicts of interest.
